# 607. Establishing Pharmacokinetic Profile of β -lactams in Critically Ill Patients on Continuous Sustained Low-efficiency Dialysis (C-SLED) 

**DOI:** 10.1093/ofid/ofac492.659

**Published:** 2022-12-15

**Authors:** Madiha Shah, Matthew Horton, Monica Donnelley

**Affiliations:** UC Davis Medical Center, Elk Grove, California; UC Davis Medical Center, Elk Grove, California; University of California Davis Medical Center, Sacramento, California

## Abstract

**Background:**

Critically ill patients have altered pharmacokinetic (PK) due to severity of illness and multi-organ failure. The lack of pharmacokinetic and pharmacodynamic data in Continuous Sustained Low-efficiency Dialysis (C-SLED) further perpetuates the uncertainty of efficacy with current antimicrobial dosing strategies. The objective of this study is to evaluate patient-specific PK of various β-lactams antibiotics in critically ill patients on C-SLED to assess if the current dosing strategy leads to therapeutic drug levels.

**Methods:**

This prospective observational cohort study was performed at an academic medical center from November 2021 to June 2022. It included patients age > 18 years and on β-lactam antibiotics while on C-SLED. Therapeutic drug monitoring (TDM) was performed by checking two levels (peak and trough). These levels were used to calculate patient-specific elimination rate constant (k), half-life, and the fraction of time above MIC (fT>MIC). Other data collected included demographics, β-lactam regimens, source of infection, microbiology data, mortality on days 7 and 30, and length of stay. Descriptive statistics were used for data analysis.

**Results:**

There were 11 patients included in the study with a median age of 55 and 82% (n = 9) of patients were male. β-lactam daily regimen was dosed as creatinine clearance (CrCl) > 50mL/min in 71.4% of patients (n = 10), CrCl 30-50 mL/min in 28.5% (n = 4), and < 30mL/min in 0% of patients. An extended infusion dosing strategy was used in 35.6% of patients (n = 5). The therapeutic drug target (50%fT > MIC for penicillins, 60%fT > MIC for cephalosporins, 40%fT > MIC for carbapenems) was achieved in 100% (n=14) of patients. Dose adjustment was not needed in all 14 cases. However, 100%fT > MIC was only achieved in 64% (n=9) patients. The median hospital length of stay was 60 days. Mortality on days 7 and 30 was 9% (n=1) and 27% (n=3), respectively.

**Conclusion:**

Preliminary analysis shows that β-lactam daily dose based on CrCl of >30mL/min leads to therapeutic drug levels in patients on C-SLED.

**Disclosures:**

**All Authors**: No reported disclosures.

